# Temporally extended successor feature neural episodic control

**DOI:** 10.1038/s41598-024-65687-w

**Published:** 2024-07-02

**Authors:** Xianchao Zhu

**Affiliations:** 1 Key Laboratory of Grain Information Processing and Control (Henan University of Technology), Ministry of Education, Zhengzhou, 450001 China; 2 Henan Key Laboratory of Grain Photoelectric Detection and Control, Henan University of Technology, Zhengzhou, 450001 China; 3 School of Artificial Intelligence and Big Data, Henan University of Technology, Zhengzhou, 450001 China

**Keywords:** Reinforcement learning, Temporal abstraction, Successor feature, Episodic control, Computational science, Computer science

## Abstract

One of the long-term goals of reinforcement learning is to build intelligent agents capable of rapidly learning and flexibly transferring skills, similar to humans and animals. In this paper, we introduce an episodic control framework based on the temporal expansion of subsequent features to achieve these goals, which we refer to as Temporally Extended Successor Feature Neural Episodic Control (TESFNEC). This method has shown impressive results in significantly improving sample efficiency and elegantly reusing previously learned strategies. Crucially, this model enhances agent training by incorporating episodic memory, significantly reducing the number of iterations required to learn the optimal policy. Furthermore, we adopt the temporal expansion of successor features a technique to capture the expected state transition dynamics of actions. This form of temporal abstraction does not entail learning a top-down hierarchy of task structures but focuses on the bottom-up combination of actions and action repetitions. Thus, our approach directly considers the temporal scope of sequences of temporally extended actions without requiring predefined or domain-specific options. Experimental results in the two-dimensional object collection environment demonstrate that the method proposed in this paper optimizes learning policies faster than baseline reinforcement learning approaches, leading to higher average returns.

## Introduction

Reinforcement learning (RL) aims to construct intelligent agents that learn through interaction with the environment to solve sequential decision tasks while maximizing the expected cumulative reward^1–4^. RL has garnered significant interest in both the research and industrial sectors, particularly when combined with deep learning methods in a framework known as deep reinforcement learning^5,5^. It finds applications in various fields related to human intelligence, such as gaming^5–12^, autonomous driving^13,14^, robotics^15,16^, software testing^17,18^, quantum control^19–21^, and more. However, existing deep reinforcement learning algorithms often require large amounts of data and millions of training steps to achieve human-level performance, which is considered a challenge due to their low sample efficiency. Therefore, researchers have been diligently finding methods to enhance the sample efficiency of deep reinforcement learning algorithms, aiming to reduce the required training data. This includes improving algorithms^22,23^, utilizing more efficient model architectures^24^, and introducing domain knowledge to expedite training^25,26^. This field is continuously evolving, and there may be more breakthroughs and innovations in the future to address these challenges.

Neural Episodic Control (NEC) is a technique proposed to address data efficiency issues^27,28^. Episodic control aims to assist Deep Reinforcement Learning (DRL) agents in making appropriate decisions by leveraging past experiences in unseen environments. The inspiration for this idea comes from the biological mechanism of the hippocampus^29^. It does not involve gradually learning representations of solutions, i.e., the expected future rewards for actions in specific situations. Instead, it directly stores observed experiences, which are the final rewards of actions, in memory. When similar problems are reencountered, the agent recalls experiences stored in memory to determine which actions will likely yield the best results^30^. Consequently, the agent can retrieve historical high-reward experiences by searching for similar cached states in episodic memory. It then estimates state values based on the retrieved similar states. Through this approach, the strategy can effectively reduce the bias between episodic state values and model-estimated state values and generalize from past high-reward scenarios.

Furthermore, solutions to reinforcement learning tasks often share structural elements, meaning that different tasks can be decomposed into a typical sequence of subtasks^31–35^. These subtasks link the time scales of raw actions occurring at each step with the time scales of broader abstract tasks, facilitating planning on these extended time scales^36–39^. Thus, temporal abstraction is the process by which agents learn the temporal structure of tasks in a way that can cut down cognitive load and enhance the generalization ability of jobs with shared structure^40–45^. One formal approach to addressing this form of abstraction is the options framework^39,46–50^. Agents using options seek to learn a set of policies related to different subtasks, along with their initiation and termination conditions. While this successfully quantifies the problem of temporal abstraction, the process can become more complex than learning simple value functions if there are no predefined, handcrafted domain-specific options^51–56^.

In this paper, we introduce a new method that combines the flexibility of sample-efficient learning with the advantages of temporal abstraction using episodic control. Specifically, this model enhances agent training by incorporating episodic memory, facilitating the learning of superior policies. It leverages episodic memory to store historical high-reward experience data. It uses this information to guide agent training, significantly reducing the number of iterations required to learn the optimal strategy. During training, the model can dynamically extract historical high-reward information from episodic memory and seamlessly integrate this information into the neural network, optimizing sample efficiency more effectively. Furthermore, we adopt the temporal expansion of successor features a technique to capture the expected state transition dynamics of actions^57–59^. This is achieved by constructing successor features on top of repeated primitive actions. This form of action abstraction does not entail learning a top-down hierarchy of task structures but focuses on the bottom-up combination of actions and repeated actions. The bottom-up approach depends on a natural idea: solution of any subproblem depends only on the solution of smaller subproblems. It uses extra memory to store the solution to sub-problems, avoids recomputation and improves performance by a huge margin. So bottom-up approach sorts the subproblems by their input size and solves them iteratively in the order of smallest to largest. In other words, when solving a particular subproblem, bottom-up approach will first solve all of the smaller subproblems its solution depends upon and store their values in extra memory. With the bottom-up approach, sometimes, there is scope to optimize the time and space complexity further. As such, our method directly considers the time horizon of temporally extended action sequences without the need for predefined or domain-specific options As a result, it reduces the number of decisions necessary to learn the optimal strategy without the need for hierarchical policy learning. Thus, our approach directly considers the temporal scope of sequences of temporally extended actions without requiring predefined or domain-specific options. Experimental results show that the method proposed in this paper optimizes learning policies faster than baseline reinforcement learning approaches, leading to higher average returns.

## Preliminaries

In this section, we develop the foundations that will help us understand the concepts, techniques, and mathematical underpinnings essential to the contributions of this paper. In particular, we introduce the key definitions and notation of the RL problem alone with episodic control and successor features.

### Reinforcement learning

The Reinforcement Learning (RL) problem is typically formalized as a Markov Decision Process (MDP), which is a key underlying assumption for much of this paper as well. A finite, discrete-time MDP is a six tuple of $\langle {\mathcal {S}}, {\mathcal {A}}, {\mathcal {R}}, {\mathcal {T}}, \gamma , \rho _0\rangle$, where ${\mathcal {S}}$ is a finite set of states, ${\mathcal {A}}$ represents a finite set of actions, ${\mathcal {R}}:{\mathcal {S}}\times {\mathcal {A}}\rightarrow {\mathbb {R}}$ is the reward function, ${\mathcal {T}}: {\mathcal {S}} \times {\mathcal {A}} \times {\mathcal {S}}\rightarrow [ 0,1 ]$ denotes a state transition dynamics, $\rho _0$ represents the initial state distribution, and $\gamma \in [0,1]$ denotes a discount factor. The behavior of agent is determined by its behavior , denoted $\pi : S \rightarrow [ 0,1 ]$, a mapping from states to probabilities of taking each of admissible original actions. The value of being in a state is determined by the state value function $V^\pi (s)=E_\pi [G_t|S_t=s]$, defined as the expected cumulative return starting from state *s*, and then following strategy $\pi$.

Similarly, the value of being in a state *s* and taking an action *a* is called the state-action value function $Q^\pi (s,a)=E_\pi [G_t|S_t=s, A_t=a]$. The Bellman equation for $Q^\pi (s,a)$ is:

1 $$\begin{aligned} Q^\pi (s,a)=\sum _{s'\in {\mathcal {S}}}\sum _{r\in {\mathcal {R}}} T(s',r|s,a)\left[ r+\sum _{a'\in {\mathcal {A}}}\pi (a'|s')Q^\pi (s',a')\right] ,\quad \forall s \in {\mathcal {S}} \end{aligned}$$

Traditionally, actions executed by agents were single-step primitive actions, meaning that the agent resampled its strategy at state $s'$ to determine the following action. Recent research has extended this action selection strategy by introducing action duration, specifying how often the chosen action is repeated before the policy is queried^60–63^. In this way, the agent possesses an action selection policy $\pi ^a:S \rightarrow A$ and an action repetition strategy $\pi ^j:S \rightarrow J$. Here, we follow the description in Ref.^61^, where *j* represents the number of action repetitions, and *J* is the set of allowed action repetition counts.

The same methods to learn standard policies can be employed to discover the action repetition policy $\pi ^j$. This paper uses a Q-learning-style update rule for learning the action repetition, as derived in Ref.^61^. This approach is related to constructing temporal extended successor features. However, there are also methods for learning the repetition policy through policy gradient learning. The optimal repetition policy $\pi ^{j*}$ can be obtained by greedily selecting from the optimum $Q^{\pi ^{j*}}(s,j, a)$, where repeated action values are derived from transitions in the environment based on the following conditions:

2 $$\begin{aligned} Q^{\pi ^{j*}}(s,j,a)=E^\pi \left[ \sum ^{j-1}_{k=0} \gamma ^k r_{t+k}+ \gamma ^j Q^\pi (s_{t+j},a^*_{t+j})|s_t=s,a,j\right] , \end{aligned}$$

where $$a^*_{t+j}=arg \max _a [Q^\pi (s_{t+j},a)]$$.

### Episodic control

Episodic memory draws from the psychological and cognitive research on human memory^64,65^ and follows the principles of instance-based decision theory^66^. Extensive research has applied episodic memory to reinforcement learning to enhance sample efficiency. For instance, in Ref.^29^, a context control method was proposed that uses episodic memory to store experiences, allowing the agent to replicate state-action sequences with high rewards. Gershman et al.^67^ utilized episodic memory to construct context reinforcement learning for state value function estimation. Lin et al.^68^ introduced a regularization term into the objective function to distill information from episodic memory into parameter models, significantly improving the performance of DQN (Deep Q-Network). Lin et al.^29^ introduced a method known as Neural Episodic Control (NEC)^27^, which employs differentiable neural dictionaries to record slowly changing state-action pairs and rapidly updated state function values. NEC corrects policies by looking up values from the context of state functions. NEC adopts two techniques to enhance the extendibility of the model. Firstly, the number of elements involved in lookups is limited to the 50 nearest neighbors, and this can be efficiently achieved using a kd-tree. Removing the least recently adopted items controls the differentiable neural dictionary (DND) size. Additionally, the values saved in the episodic memory pool are N-step Q-value estimates:

3 $$\begin{aligned} Q^{(N)}(s_t,a_t)=E \left[ \sum ^{N-1}_{i=0}\gamma ^i r_{t+i}+ \gamma ^N Q(s_{t+N},a')\right] \end{aligned}$$

During learning, Q-learning^69^ is utilized to update the Q-values in the episodic memory pool for the keys already found in the DND. This process, carried out by $Q_i \leftarrow Q_i+ \alpha (Q^{(N)}(s,a)-Q_i)$, helps refine and update the Q-value estimates for the stored experiences, thereby contributing to the learning process.

### Successor features

Successor features (SF) are based on representing the value function in a way that separates reward information from the environmental state transition matrix^57,58,70^. Successor features assume that rewards are a linear combination of characteristics $\varvec{\phi }_{\textbf{t}}=\phi (s_t,a_t,s_{t+1}) \in R^n$, where this combination depends on state transitions and a weighted vector $\varvec{\omega } \in R^n:r_t=\varvec{\phi }_{\textbf{t}}^T\varvec{\omega }$. Here, the features describe critical information about states, which are used to evaluate them using the reward function in a low-dimensional representation. Consequently, the Q-function can be reformulated as:

4 $$\begin{aligned} Q^\pi (s_t,a_t)=E^\pi \left[ \sum ^\infty _{i=t} \gamma ^{i-t}r_i \right] =E^\pi \left[ \sum ^\infty _{i=t} \gamma ^{i-t}\varvec{\phi }_{\textbf{i}}^T\varvec{\omega }\right] =\varvec{\psi }^\pi (s_t,a_t)^T\varvec{\omega } \end{aligned}$$

where $\varvec{\psi }^\pi (s_t,a_t)$ represents the successor features of state-action pair $(s_t,a_t)$ under strategy $\pi$.

## Temporally extended successor feature neural episodic control

In this section, we introduce Temporally Extended Successor Feature Neural Episodic Control (TESFNEC) to extend SFNEC to learn temporally extended successor features $\varvec{\psi } \in R^n$ in position scalar state-action values. Like SFNEC, TESFNEC learns the time-extended successor feature values for an repetitive action of *j*, $\varvec{\psi }^N_j$:

5 $$\begin{aligned} \varvec{\psi }^N_j(s_t,a_t)&=E\left[ \sum ^{N-1}_{i=0} \gamma ^i \varvec{\phi }^j_{t+i}+\gamma \max _{a'}\varvec{\psi }^N_j(s_{t+N},a') \right] \nonumber \\&=E\left[ \sum ^{N-1}_{i=0} \gamma ^i [E^\pi \left[ \sum ^j_{k=0}(\gamma \mathbf {T_a})^k\right] +E^\pi [(\gamma \mathbf {T_a})^j]({\mathbb {I}}-\gamma {\textbf{T}}^\pi )^{-1}\right] +\gamma \max _{a'}\varvec{\psi }^N_j(s_{t+N},a')] , \end{aligned}$$

where ${\mathbb {I}}$ is the indicator function. Compared to the SFNEC method, the representation used in our proposed method is biased based on the action space, revealing the extended transitions caused by repeatedly taking the same action. This results in representations of distant states that notably differ as the discount factor $\gamma$ changes.

To use SF for discovering action repetitions, the first step is to sample action from the policy $\pi ^a$ such that $\varvec{\psi }^N_j(s,a,s',j)$ now specify two parameters: s and a. Action repetition is then the max operation of $\varvec{\psi }^N_j(s,a,s',j)R$, where *R* is the learned reward vector. Traditional successor features are learned through temporal difference learning, with state occupancy as the accumulated reward, and the reward weights *R* are learned by online supervised learning based on expected cumulative rewards and practical rewards received. Therefore, typical temporal difference learning rules can be used to learn temporal extended successor features:

6 $$\begin{aligned}{}&\varvec{\psi }^N_j(s,a,s',j) \leftarrow ^a \varvec{\psi }^N_j(s,a,s',j)+ E^\pi \left[ \sum ^{j-1}_{k=0} {\mathbb {I}}(s_k=s) +\gamma ^j \varvec{\psi }^N(s,a^*,s')-\varvec{\psi }^N_j(s,a,s',j)\right] . \end{aligned}$$

Moreover, to execute a lookup using the TESFNEC method, we adopt the following equation:

7 $$\begin{aligned} \psi ^j(s_t,a)=\sum _l \frac{q(s_t,s_l)}{\sum _i q(s_t,s_i) * \psi ^j_l}, \end{aligned}$$

where $\psi ^j_l$ corresponds to an anteriorly stored $\psi ^j_l$-value for state $s_l$ in episodic memory, and *q* is the kernel adopted to calculate an index of similarity between the state of query $s_t$ and sates in episodic memory $s_l$. In this paper, we use the state vector $s_t$ as the key value in the episodic memory in our experiments. Like SFNEC, we restrict the memory elements used during lookups to the nearest features, such as the nearest 50. Likewise, we also employ the inverse distance kernel used in^27^: $q(s_t,s_l)=\frac{1}{\left\| s_t-s_l \right\| ^2_2+\delta }$.

During training processing, $\psi ^j$ values are updated after observing *N* transitions. When the $\psi ^j$ value of state-action pair (*s*, *a*) does not exist in the episodic memory, the *N*-step estimate calculated using Equation (8) is inserted into the corresponding DND for action *a*. On the other hand, for $\psi ^j$ values already in episodic memory, the following formula is used for updates:

8 $$\begin{aligned} \psi ^j_l \leftarrow \psi ^j_l +\alpha (\varvec{\psi }^N_j(s,a)-\psi ^j_l), \end{aligned}$$

where $\alpha$ is the learning rate.

The pseudo-code of the Temporally Extended Successor Feature Neural Episodic Control (TESFNEC) is as shown in Algorithm 1.

**Algorithm 1:**
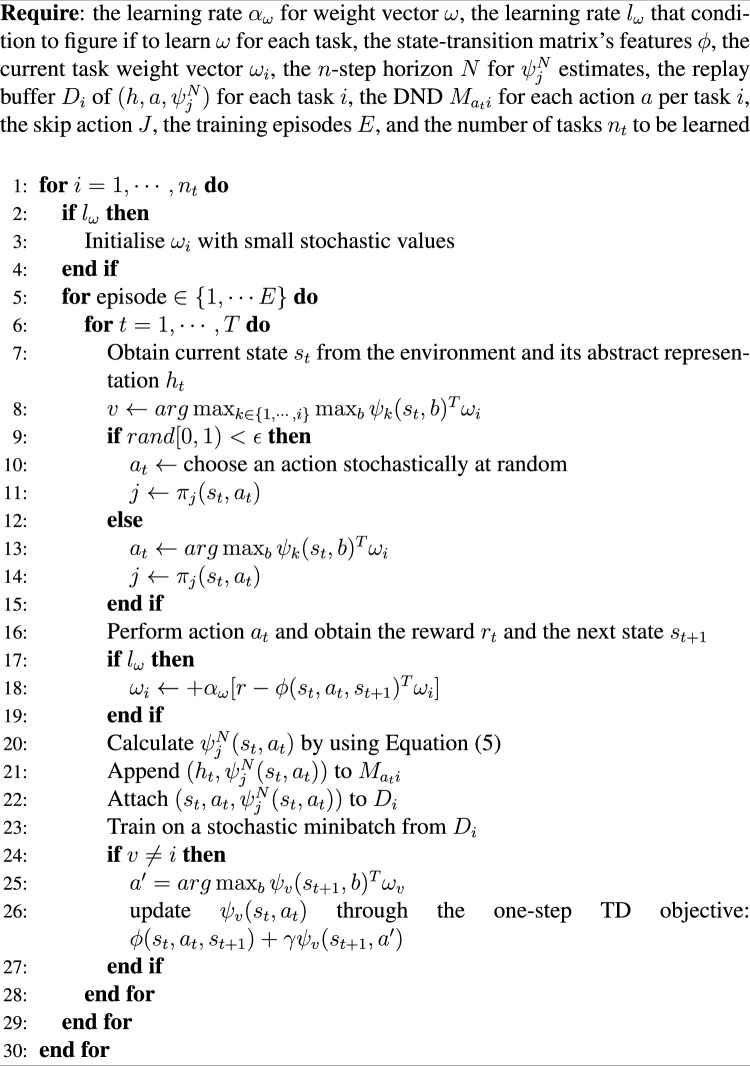
Temporally Extended Successor Feature Neural Episodic Control (TESFNEC)

## Experiment

**Figure 1:**
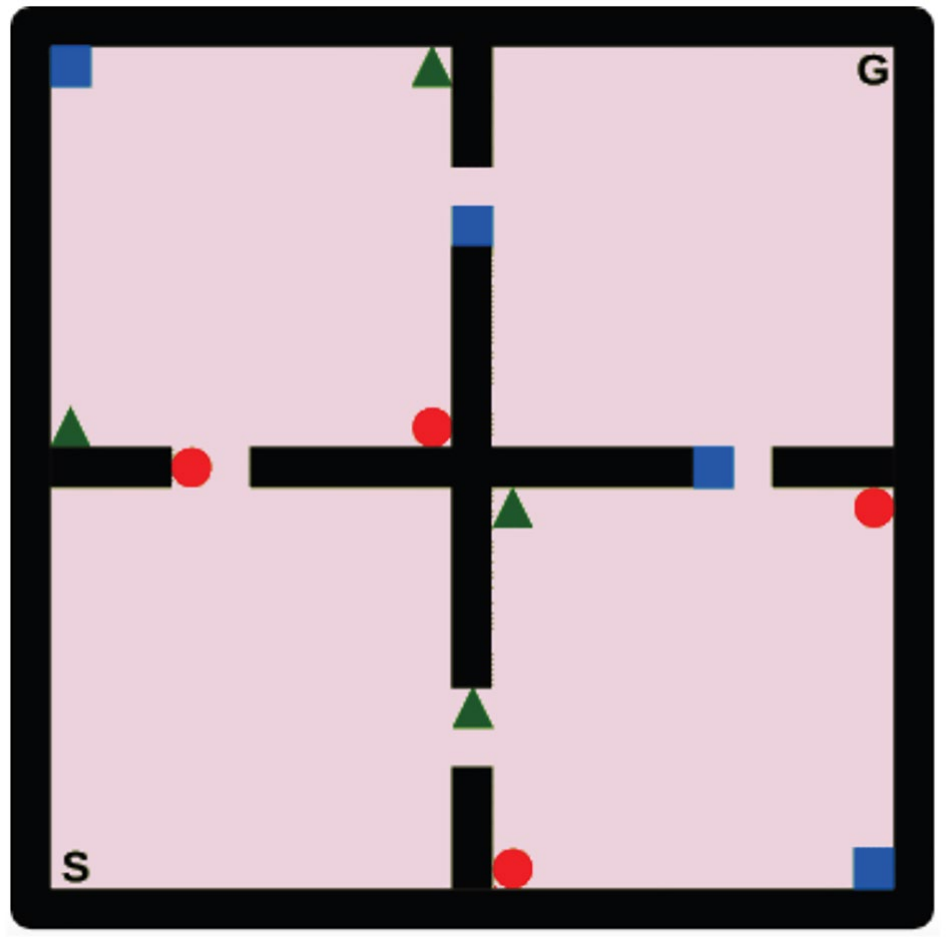
Two-dimensional object collection environment presented by Barreto et al.^57^.

The performance of the presented TESFNEC method is evaluated in the two-dimensional object collection domain as presented by Barreto et al.^57^ (Fig. 1). This environment consists of four rooms, with the starting position located in the bottom-left corner, represented as ”S,” and the target location in the top-right corner, marked as ”G.” Within each room, there are multiple objects belonging to three categories: circles, squares, and triangles. The objective is to navigate from the start to the target position while picking up things to maximize expected cumulative rewards. It is necessary to linearly decompose the rewards into characteristics and weights, as represented by $r_t=\varvec{\phi }_{\textbf{t}}^T\varvec{\omega }$. These features describe the class of the collected object and whether the target state is reached in binary form: $\phi \in \{ 0,1\}^4$. Different environmental tasks are defined by setting the weight vector $\varvec{\omega }$ accordingly.

**Figure 2:**
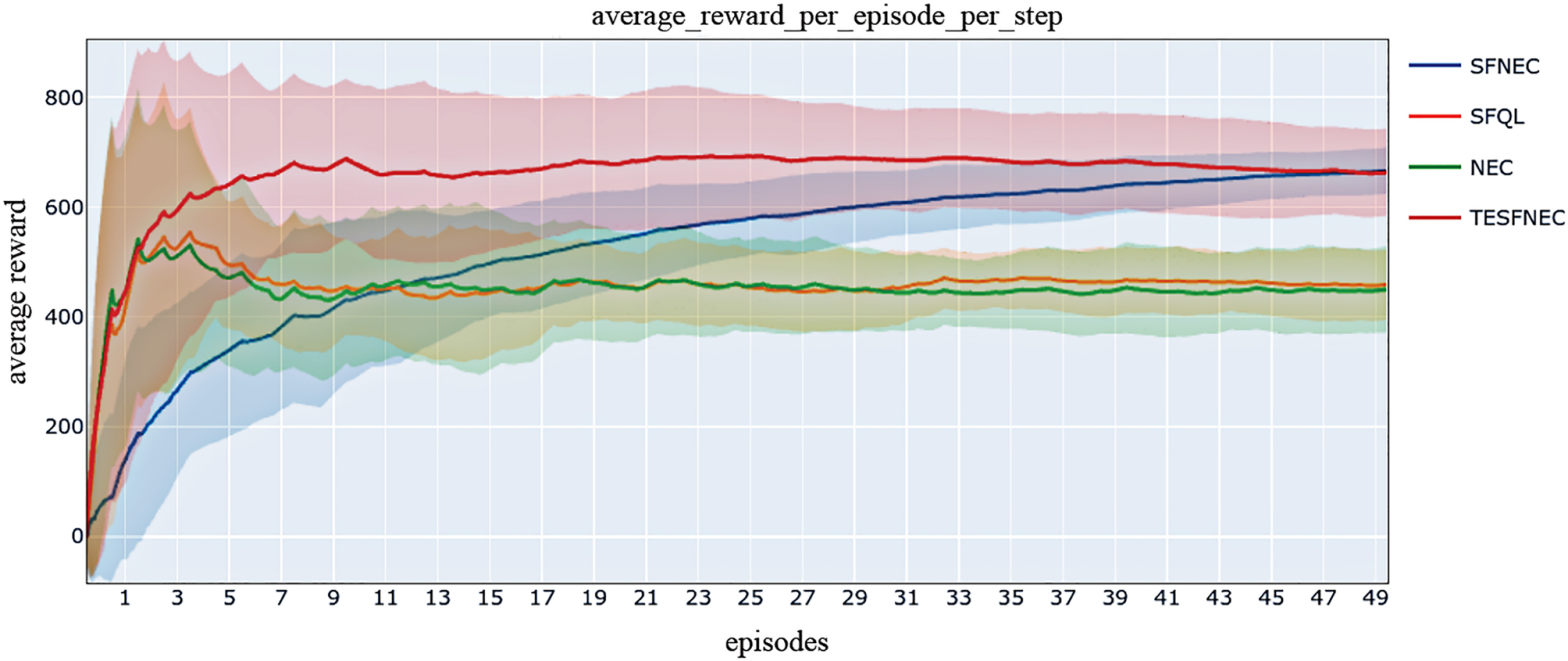
Comparison of the average reward obtained by different methods on the two-dimensional object collection environment.

To exhibit good manifestation, the agent faces a battery of tasks, each of which is a disparate instance of weight vector w, to maximize the cumulative reward sum. Generally, we adopt the same environment setting as in Barreto et al.^57^. We compared the proposed TESFNEC method with SFNEC, SFQL, and NEC methods. The comparison is based on the average returns for each task across ten runs (Fig. 2). The TESFNEC method, which combines temporally extended successor features with episodic control techniques, outperforms SFNEC, SFQL, and NEC methods regarding task performance. This is likely because TESFNEC leverages the learning speed of scenario control for each task and combines it with the powerful flexibility and abstract representation capabilities provided by temporal extended successor features.

**Figure 3:**
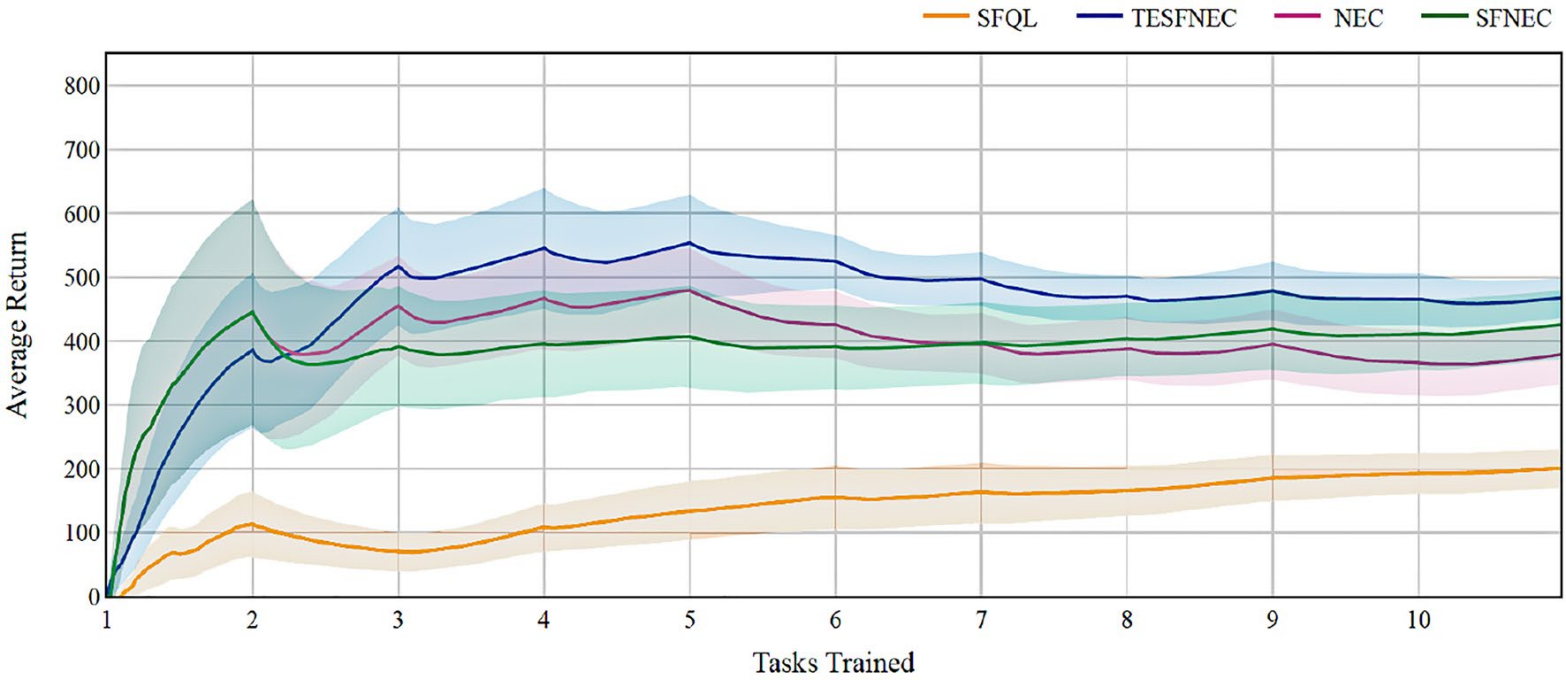
Comparison of the average reward obtained by different methods on the two-dimensional object collection environment while learning the reward weighted vector $\varvec{\omega }$.

Furthermore, we develop experiments where the reward weighted vector $\varvec{\omega }$ is not provided to agents; rather, it is approximated while interacting with the environment for methods that need $\varvec{\omega }$ i.e., SFNEC and TESFNEC. As shown in Fig. 3, we observed a reduction in the average return across all methods that rely on $\varvec{\omega }$.

## Conclusion

In this paper, we introduce a novel approach that combines the flexibility of sample-efficient learning with the advantages of temporal abstraction using episodic control. Specifically, this model enhances agent training by incorporating episodic memory, facilitating the learning of superior policies. It leverages episodic memory to guide agent training, significantly reducing the number of iterations required to learn the optimal policy. During training, the model can dynamically extract historical high-reward information from episodic memory and seamlessly integrate this information into the neural network, optimizing sample utilization more effectively. Furthermore, we adopt the temporal expansion of successor features technique to capture the expected state transition dynamics of time-extended actions. This form of action abstraction does not entail learning a top-down hierarchical task structure but focuses on the bottom-up combination of actions and action repetitions. As a result, it reduces the number of decisions necessary to learn the optimal strategy without the need for hierarchical policy learning. Thus, our approach directly considers the temporal scope of sequences of time-extended actions without requiring predefined or domain-specific options. Experimental results demonstrate that our presented method optimizes learning policies faster than baseline reinforcement learning methods, leading to higher average returns.

## Data Availability

Our approach is primarily based on two research works, Successor Feature Neural Episode Control (SFNEC) and Temporally Extended Successor Representations (t-SR), which are two representative methods for temporal abstraction. The source code for SFNEC and t-SR can be referred to in https://github.com/mjsargent and https://gitlab.inria.fr/robotlearn/sfnec, respectively.
